# A More Robust Gut Microbiota in Calorie-Restricted Mice Is Associated with Attenuated Intestinal Injury Caused by the Chemotherapy Drug Cyclophosphamide

**DOI:** 10.1128/mBio.02903-18

**Published:** 2019-03-12

**Authors:** Tao Liu, Yanqiu Wu, Linghua Wang, Xiaoyan Pang, Liping Zhao, Huijuan Yuan, Chenhong Zhang

**Affiliations:** a State Key Laboratory of Microbial Metabolism, Joint International Research Laboratory of Metabolic and Developmental Sciences, and School of Life Sciences and Biotechnology, Shanghai Jiao Tong University, Shanghai, China; b Department of Biochemistry and Microbiology and New Jersey Institute for Food, Nutrition, and Health, School of Environmental and Biological Sciences, Rutgers University, New Brunswick, New Jersey, USA; c Henan Provincial People’s Hospital, Zhengzhou, Henan Province, China; New York University; Danone France; Institute of Digestive Diseases, Prince of Wales Hospital, The Chinese University of Hong Kong

**Keywords:** cyclophosphamide, *Lactobacillus*, calorie-restricted, gut microbiota, mucositis

## Abstract

Cyclophosphamide (CTX) is widely used in cancer chemotherapy, but it often induces mucositis, in which the disruption of the gut microbiota might play a pivotal role. Whether the manipulation of the gut microbiota can be used as a strategy to improve CTX-induced mucositis remains to be studied. Here we observed the effects of a 4-week calorie restriction (CR) on CTX-induced mucositis. Compared with *ad libitum*-fed mice, CR mice showed significantly less mucositis in response to CTX, including lower intestinal permeability, less bacterial translocation, higher number of epithelial stem cells, and less epithelium damage. CTX induced significant shifts of the gut microbiota of the gut microbiota in *ad libitum*-fed control mice. In contrast, CR mice showed no significant change in their gut microbiota in responding to CTX treatment. CR significantly enriched the gut microbiota in *Lactobacillus* and *Lachnospiraceae* which are known to mitigate inflammation and improve gut barrier function. These findings suggest that CR remodeled gut microbiota is more robust and may contribute to attenuate the side effects of cyclophosphamide, which supports the concept that cancer chemotherapy would benefit from strategies targeting the gut microbiota.

## Abstract (precis)

Improving the gut microbiota via calorie restriction is beneficial for human health. Our findings showed differential responses between calorie-restricted mice and *ad libitum*-fed mice. Compared with the *ad libitum*-fed mice, the calorie-restricted mice were less susceptible to cyclophosphamide side effects otherwise observed on the gut integrity and its microbiota. These results show the potential benefits of manipulating the gut microbiota with CR prior to cancer chemotherapy.

## INTRODUCTION

Cyclophosphamide (CTX), the most widely used chemotherapy drug, can induce tumor cell death because of its genotoxicity and cytotoxicity (1–4). However, the toxicity of CTX can nondiscriminatorily affect tumor cell and other rapidly dividing healthy cells, such as the intestinal stem cells (5–8). Thus, CTX can increase the intestinal permeability and induce the development of mucositis by damaging the normal (healthy) intestinal epithelium (9, 10). There are still no effective ways to avoid these side effects in the clinical treatment of cancer with CTX (11).

The normal gut microbiota plays an important role in maintaining the function of the gut barrier, and dysbiosis of the gut microbiota can induce the damage of the intestinal epithelium (12–14). For example, high-fat diet or dextran sulfate sodium induced overgrowth of sulfate-reducing bacteria *Desulfovibrio* spp. that damaged the gut barrier (15, 16). In contrast, increasing the abundance of protective bacteria, such as *Bifidobacterium* spp. and *Lactobacilli* spp., was associated with improvement of the intestinal barrier function by inhibiting the adhesion of pathogenic bacteria to the intestinal wall or modulating epidermal growth factor receptor-mediated intracellular signaling (17–21). Previous studies showed that chemotherapy can shift the structure of gut microbiota, such as significant enrichment of the Clostridium leptum in mice and reduction of the numbers of anaerobic bacteria in the human gut (22, 23). Moreover, diminishment of the intestinal microbiota by antibiotic treatment aggravated the toxicity of chemotherapy to the intestinal epithelium (22). This result suggests that the manipulation of the gut microbiota may provide a promising strategy to counteract the side effect of CTX.

Diet is the most important factor to modulate the gut microbiota (24, 25). Not only the composition of the diet but also the quantities of food consumed can modify the gut microbiota (26). Previous studies showed that calorie restriction (CR) can significantly shift the gut microbiota in both humans and mice (27, 28). One major change in CR mice was the enrichment of protective bacteria such as *Lactobacillus* spp. (26). Our recent study identified a strain of Lactobacillus murinus that thrived in CR mice and contributed to the protection of the gut barrier and attenuation of ageing-associated inflammation (29). CR or short-term starvation has been shown to mitigate the CTX-caused oxidative stress in the mouse model (30–32). However, it was still unclear whether the CR-modulated gut microbiota contributes to alleviation of side effects from chemotherapy.

To investigate this question, we administered CTX to C57BL/6J mice after 4 weeks of CR, and the mice that were fed *ad libitum* were used as control group. We found that the CR-modulated gut microbiota was associated with protection of the intestinal barrier and epithelium from damage caused by CTX. These results suggest that manipulating the structure of the gut microbiota may be used as a strategy to alleviate the side effects of CTX.

## RESULTS

### Calorie restriction protected mice from the toxicity of cyclophosphamide observed in the intestinal epithelial cells.

We randomized 8-week-old male C57BL/6J mice into two groups (*n* = 35 per group): fed *ad libitum* with normal chow diet (*ad libitum* group) or fed with 70% normal chow of the *ad libitum* (CR group) (see Fig. S1 in the supplemental material). The body weight of mice in the CR group was significantly decreased and stayed stable after 14 days (Fig. S2B). Hematoxylin-eosin staining of the distal colon showed that the structure of the intestinal epithelium had no significant differences between the CR and *ad libitum* group at 28 days. However, the serum level of lipopolysaccharide (LPS)-binding protein (LBP) (33), which reflects the bacterial antigen load in host blood, was significantly lower in the CR group (Fig. S2C to E).

10.1128/mBio.02903-18.1 FIG S1 Study design. Download FIG S1, PDF file, 0.1 MB. Copyright © 2019 Liu et al. 2019 Liu et al. This content is distributed under the terms of the Creative Commons Attribution 4.0 International license.

10.1128/mBio.02903-18.2 FIG S2 CR reduced body weight, food intake, and LBP in the serum. (A) Food intake. (B) Body weight. (C) Concentration of lipopolysaccharide-binding protein (LBP) in serum after CTX injection. (D) Histological scores of colon epithelium, based on the inflammation and mucosal damage. (E) Histological section by HE staining. All data are shown as means ± SEM. Mann-Whitney test was used to analyze variation between *ad libitum* and CR. *, *P* < 0.05; **, *P* < 0.01; ***, *P* < 0.001. Download FIG S2, PDF file, 0.4 MB. Copyright © 2019 Liu et al. 2019 Liu et al. This content is distributed under the terms of the Creative Commons Attribution 4.0 International license.

After 4 weeks, half of the mice in each group were intraperitoneally injected with CTX (100 mg/kg body weight), and the other half were injected with normal saline (NS) as the control (Fig. S1). CTX induced significant decrease of food consumption in the *ad libitum*-fed mice, but not in CR mice (Fig. S3A). CTX showed no significant effects on body weight in both CR mice and *ad libitum*-fed mice (Fig. S3B).

10.1128/mBio.02903-18.3 FIG S3 Effects of CTX on food intake, body weight, and spleen body ratio in *ad libitum*-fed and CR mice. (A) Body weight. (B) Food intake. All data are shown as means ± SEM. Two-way ANOVA was used to analyze variation at the same time point. Values that are significantly different in the NS group and CTX group with the same diet are indicated (#). *, *P* < 0.05; **, *P* < 0.01; ***, *P* < 0.001. Download FIG S3, PDF file, 0.1 MB. Copyright © 2019 Liu et al. 2019 Liu et al. This content is distributed under the terms of the Creative Commons Attribution 4.0 International license.

**FIG 1:**
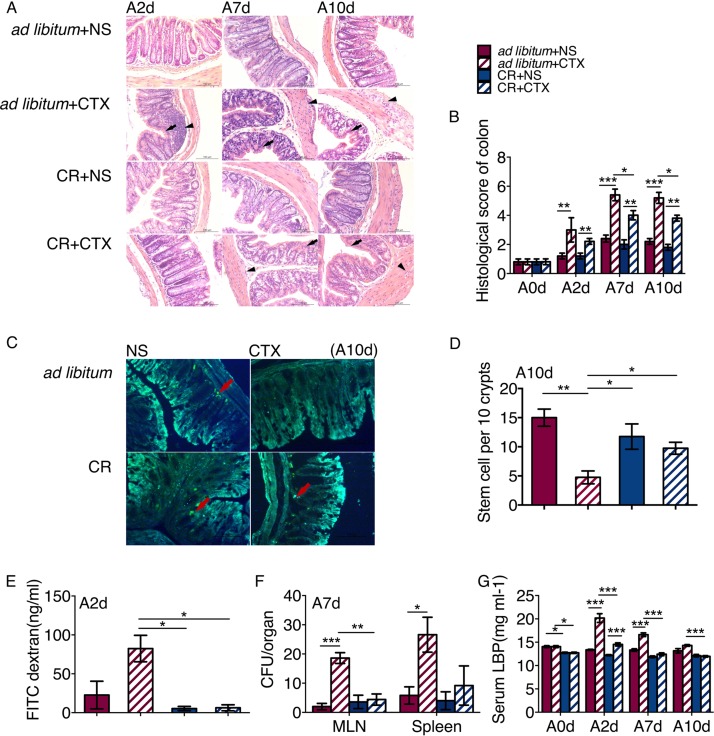
CR reduced the intestinal permeability and protected the epithelium from CTX. (A) Histological section by HE staining. (B) Histological scores of colon epithelium based on inflammation and mucosal damage. For each group, there were five mice (*n* = 5) every day. (C) BrdU staining of colon slices. Red arrows point to stem cells. (D) Number of ISC per 10 crypts. For each group, *n* = 5. (E) Concentration of FITC in serum at 2 days CTX injection. (F) Number of bacteria (CFU) in the MLN and spleen. (G) Concentration of lipopolysaccharide-binding protein (LBP) in serum after CTX injection. The data are shown as means ± SEM (error bars). Two-way ANOVA was used to analyze variation at the same time point. Values that are statistically significantly different are indicated by bars and asterisks as follows: *, *P* < 0.05; **, *P* < 0.01; ***, *P* < 0.001.

We evaluated the effect of CR on protecting intestinal epithelium from the side effects of CTX treatment in mice. From the 2nd day of CTX injection, CTX induced inflammatory infiltration and epithelium injury in both CR and *ad libitum*-fed mice (Fig. 1A and B), but the histological score was significantly higher in the *ad libitum*+CTX group than in the CR+CTX group. Moreover, although the intestinal stem cells (ISC) in the colon were damaged by CTX in both CR and *ad libitum-*fed mice (Fig. 1C), the number of ISC was significantly higher in the CR+CTX mice than in the *ad libitum*+CTX mice (Fig. 1D).

To detect the effect of CTX on intestinal permeability, we orally administered 4,000 Da fluorescein isothiocyanate (FITC)- labeled dextran to the mice and then measured translocation of fluorescence into plasma. The concentration of FITC in serum was significantly higher in the *ad libitum*+CTX group than in the CR+CTX group after 2 days of CTX injection (Fig. 1E). Seven days after CTX injection, we were able to detect marked bacterial growth in the mesenteric lymph nodes (MLN) and spleen of the *ad libitum*+CTX group compared to the *ad libitum*+NS mice, while the bacterial load was much lower in both the CR+CTX and CR+NS groups (Fig. 1F). Moreover, we found that LBP was significantly increased after CTX injection in both CR and *ad libitum*-fed mice. However, the concentration of LBP in serum was significantly lower in the CR+CTX group than in the *ad libitum*+CTX group (Fig. 1G). The decreased translocation of gut bacteria and bacterial antigen reflect the protection of gut barrier function by CR (22).

Taken together, these results suggest that the detrimental effects of CTX on the intestinal epithelium and gut barrier function were attenuated by CR.

### The structure of microbiota in calorie-restricted mice was more robust against cyclophosphamide treatment-induced changes.

**FIG 2:**
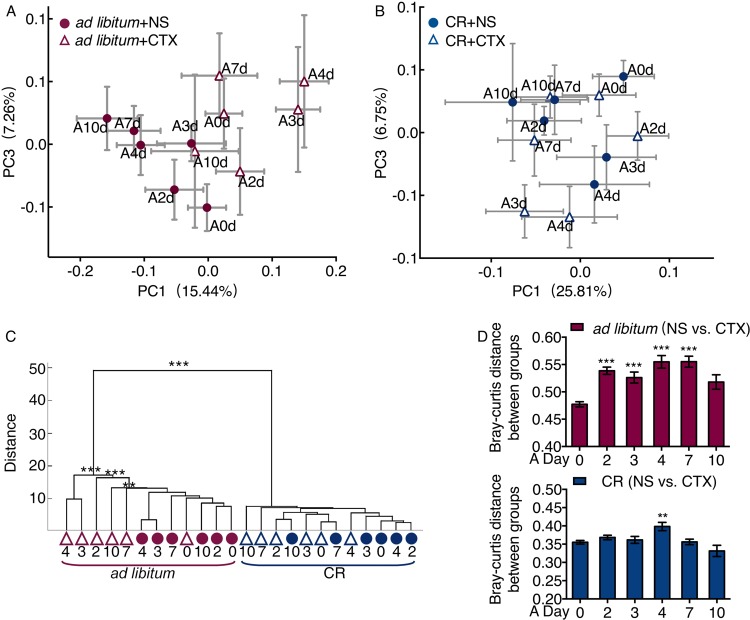
The microbiota of the mice in the CR group were much more stable than the microbiota of the mice in the *ad libitum*-fed group treated with CTX. (A and B) Variation of gut microbiota structure of *ad libitum* group and CR group after CTX injection along PC1 and PC2 of PCoA based on the Bray-Curtis distance. (C) Clustering of gut microbiota based on distances between different groups calculated by multivariate analysis of variance test of the first 35 PCs of Bray-Curtis PCoA data. (D) Bray-Curtis distance between CTX treatment and the NS control and one-way ANOVA test were used. *, *P* < 0.05; **, *P* < 0.01; ***, *P* < 0.001.

Our previous studies showed that the CR mice had a unique gut microbiota dominated by potentially beneficial bacteria such as *Lactobacillus* spp. (27). In the current study, the overall structure of the gut microbiota in CR mice was significantly shifted after 4 weeks of CR, as shown by principal-coordinate analysis (PCoA) based on Bray-Curtis distance (Fig. S4A, *P* < 0.001 in permutation multivariate analysis of variance [PERMANOVA], 9,999 permutations). In *ad libitum*-fed mice, the gut microbiota of the CTX group was clearly separated from the NS group after CTX injection (Fig. 2A and C). There was no significant difference between the CR+NS and CR+CTX groups (Fig. 2B and C). The structure of gut microbiota showed significant differences between the CTX and NS group from the third to seventh day after CTX injection in the *ad libitum*-fed group (Fig. 2C; see also Table S1 in the supplemental material). In CR mice, the CTX treatment showed smaller impact on their gut microbiota (Fig. 2D).

10.1128/mBio.02903-18.7 TABLE S1 Effects of microbiota structure by CTX. PERMANOVA was used. Download Table S1, PDF file, 0.1 MB. Copyright © 2019 Liu et al. 2019 Liu et al. This content is distributed under the terms of the Creative Commons Attribution 4.0 International license.

These results suggest that CTX could induce significant change of the microbiota structure in the *ad libitum*-fed group, but the gut microbiota in the CR group were more stable than the gut microbiota in the *ad libitum* group in response to CTX effects.

### Specific phylotypes of the gut microbiota were modulated by calorie restriction and cyclophosphamide treatment.

**FIG 3:**
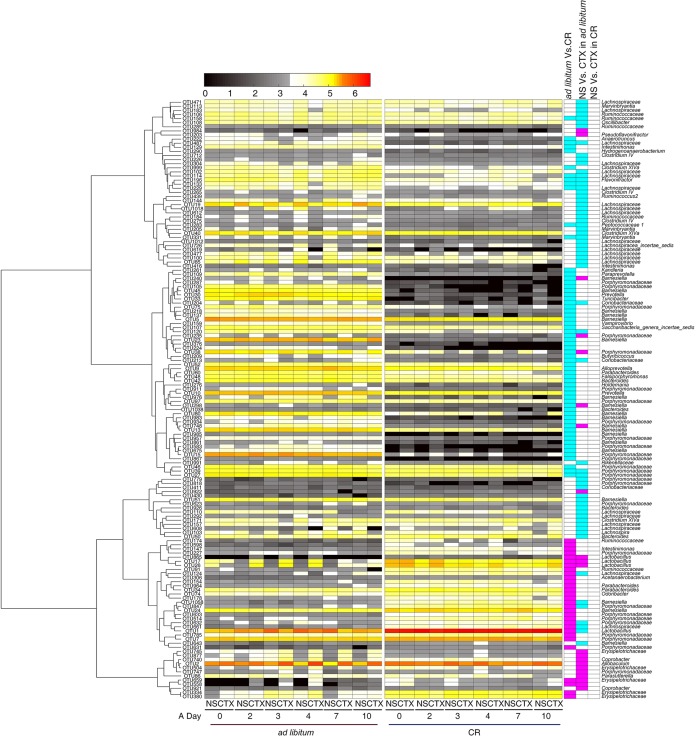
RDA-derived key phylotypes of the *ad libitum*-fed group responding to CR and CTX effects. The heat map colors of the spots in the panel represent the mean relative abundance (log-transformed) values of the OTUs in each group. The values on the color bar are the relative abundance indicated by the corresponding color. OTUs are arrayed by the Spearman correlation cluster. The key comparisons and direction of changes are summarized in three columns to the right of the heat map. A two-sided Mann-Whitney test was used for analysis. The FDR of <0.05 are shown with blue and pink box. The “*ad libitum* Vs. CR” column compares CR+NS mice to *ad libitum*+NS mice at 28d. A blue box indicates that the OTU was significantly lower in CR+NS mice, and a pink box indicates that it was significantly higher. The “NS Vs. CTX in *ad libitum*” column compares *ad libitum*+CTX mice to the *ad libitum*+NS mice. A blue box indicates that the OTU was significantly decreased by CTX, and a pink box indicates that the OTU was significantly increased by CTX. The “NS Vs. CTX in CR” column compares CR+CTX mice to the CR+NS mice.

On the basis of the results of our redundancy analysis (RDA), we identified 79 operational taxonomic units (OTUs) that were significantly changed in CR mice compared to *ad libitum*-fed mice after 4 weeks of CR (Fig. S4B). Compared to *ad libitum*-fed mice, OTU1 that belongs to the genus *Lactobacillus* became the predominant bacteria in CR mice, and 26 other OTUs were also significantly enriched in the CR group. In contrast, 53 OTUs were decreased in the CR group, most of which were phylotypes from the family *Porphyromonadaceae* (Fig. 3).

10.1128/mBio.02903-18.4 FIG S4 Specific microbiota structure built up by the CR. (A) Variation of gut microbiota structure along PC1 and PC2 based on the Bray-Curtis distance. (B) Biplot of the RDA (redundancy analysis) based on the relative abundances of the OTUs between the *ad libitum* and the CR mice. OTUs have at least 47.7% of the variability in their values explained by the canonical axis. The *P* value of the Monte-Carlo permutation test is shown at the top left corner of the graph. *n* = 35 in each group. Download FIG S4, PDF file, 0.1 MB. Copyright © 2019 Liu et al. 2019 Liu et al. This content is distributed under the terms of the Creative Commons Attribution 4.0 International license.

After injecting CTX into the mice, we identified 83 OTUs that were shifted in responding to the CTX stress in *ad libitum*-fed mice (Fig. 3 and Fig. S5). Twenty-one OTUs were increased and 62 OTUs were decreased in response to CTX. None of these OTUs were significantly affected by CTX in CR mice (Fig. 3). Among the 83 OTUs, 29 OTUs were also significantly different between CR and *ad libitum*-fed groups at 28 days. Six OTUs were enriched in CR mice and reduced by CTX in the *ad libitum*-fed group. These OTUs belonged to the *Lachnospiraceae* (2 OTUs), *Porphyromonadaceae* (2 OTUs), and *Lactobacillus* (1 OTU). However, five OTUs that belong to *Porphyromonadaceae* were reduced in CR mice and increased by CTX in the *ad libitum-*fed group (Fig. 3). On the other hand, 54 OTUs were affected only by CTX. For example, 11 OTUs were enriched by CTX, and all these OTUs belong to *Erysipelotrichaceae, Porphyromonadaceae*, and *Sutterellaceae*. However, 43 OTUs were reduced by CTX, most of which were species from the family *Lachnospiraceae* and *Ruminococcaceae*, whose members contained butyrate-producing bacteria (34).

10.1128/mBio.02903-18.5 FIG S5 Biplot of the RDA (redundancy analysis) based on the relative abundance of the OTUs between the *ad libitum*+NS group and *ad libitum*+CTX group. Download FIG S5, PDF file, 0.1 MB. Copyright © 2019 Liu et al. 2019 Liu et al. This content is distributed under the terms of the Creative Commons Attribution 4.0 International license.

### Key phylotypes showed guild-like response to calorie restriction and cyclophosphamide treatment.

**FIG 4:**
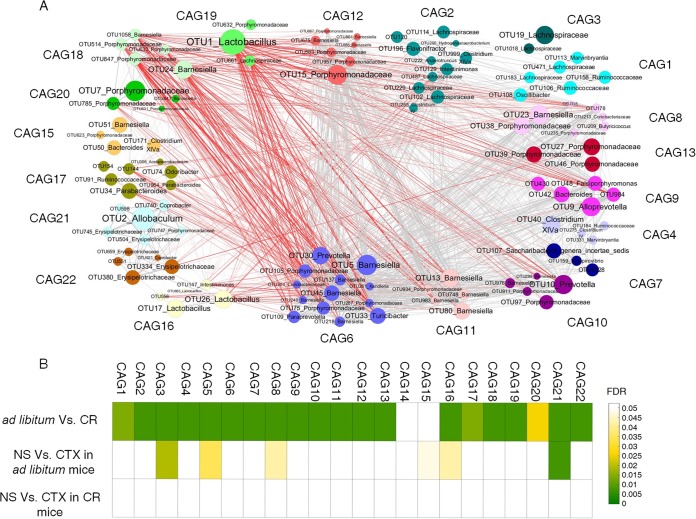
Coabundance groups of all the key OTUs in mice. (A) OTU-level network diagram of 146 key OTUs responding to the effects of CTX and different diet. The size of the node indicates the mean abundance of each OTU. Lines between nodes represent correlations between the nodes they connect, with the line width indicating correlation magnitude and color for correlation (red for a negative correlation, gray for a positive correlation). Only lines corresponding to correlations with a magnitude greater than 0.5 are drawn. OTUs are grouped into 22 CAGs by permutation multivariate analysis of variance (PERMANOVA) when *P < *0.05. (B) Heat map of the FDR value for CAGs, indicating the changes induced by diet or CTX stress. The tests of significance were performed using a two-sided Mann-Whitney test, and the calculation of FDR was performed using the procedure originally introduced by Benjamini and Hochberg.

Bacteria may function together as a functional group called guilds (35). Coabundance analysis may capture such structures in gut microbiota (36). We then constructed a coabundance network to illustrate the potential interaction among the 146 nonredundant OTUs that were significantly affected by CTX or CR. These OTUs were clustered into 22 coabundance groups (CAGs) based on Spearman correlation analysis (Fig. 4A).

Six CAGs were significantly different between the *ad libitum*+CTX group and *ad libitum*+NS group (Fig. 4B). For example, CAG3, which was mainly composed of the OTUs from *Lachnospiraceae,* was significantly decreased by CTX. However, CAG8, CAG15, CAG16, and CAG21 were increased by CTX (Fig. S6). Twenty CAGs were significantly shifted by CR (Fig. 4B). In contrast to *ad libitum*-fed mice, all of the CAGs were not significantly changed by CTX in CR mice. These results suggested that CR can significantly reduce the modulating effects of CTX on the gut microbiota.

10.1128/mBio.02903-18.6 FIG S6 Relative abundance of each CAG. Data in the plot represent the abundance of all OTUs in each CAG from each sample, which were then visualized by mean ± SEM. Two-way ANOVA test was used to analyze variation at the different time points. *, *P* < 0.05; **, *P* < 0.01; ***, *P* < 0.001. Download FIG S6, PDF file, 0.2 MB. Copyright © 2019 Liu et al. 2019 Liu et al. This content is distributed under the terms of the Creative Commons Attribution 4.0 International license.

CAG8, which was composed of OTUs belonging to *Porphyromonadaceae*, was significantly enriched by CTX but reduced by CR. In contrast, four CAGs were significantly negatively correlated with CAG8, including CAG19, which mainly contained OTU1 in *Lactobacillus* spp.

## DISCUSSION

Our study shows that CR attenuated the mucositis caused by CTX, including intestinal permeability, bacterial translocation, and epithelium damage, while at the same time, CR changed the gut microbiota structure which became more robust under the later CTX treatment.

CTX exacerbated the dysbiotic state of the gut microbiota, which not only reduces the effects of tumor suppression but also decreases the quality of life of the patients (37, 38). Clinically, more and more pieces of evidence focused on the role of gut microbiota in chemotherapy. For instance, recently probiotics were used in combination with chemotherapy to alleviate the side effects, such as diarrhea (39). CR or starvation could reduce the toxicity of CTX (32, 40, 41). Starvation can be efficient in protecting the host from oxidative stress induced by CTX in both rats and mice (32, 41). However, the mechanism of such protective effects are still unclear. In our current work, we focus on CR modeled gut microbiota to mitigate the side effects of the chemotherapy drug.

Our findings suggest that the gut microbiota structure in CR mice was linked to reduced CTX-induced damage. A significant change in the overall structure of the gut microbiota was observed in CTX-treated mice in our work. Interestingly, CR mice maintained their gut microbiota structure despite the treatment effects of CTX, showing a significant robustness against perturbations. Accumulating evidence suggests that robust gut microbiota is tightly linked with the host health (42, 43). A previous study of mice showed that greater perturbation on the gut microbiota by antibiotic treatment increased higher susceptibility to intestinal colonization, more disruption in the microbiota, and more severe intestinal pathology than mice whose gut microbiota were not perturbed by Salmonella enterica serovar Typhimurium (44). These results suggest that more stable gut microbiota structure may bring more benefits to the host against disorders induced by various perturbations such as chemotherapy.

In addition to the more robust overall structure of the gut microbiota, specific bacterial phylotypes or their “guilds” may play a more significant role in mediating the CR-induced protective effects. In the current study, we also detected similar modulation of gut microbiota by CR, especially significant increase of *Lactobacillus* spp. (27). The gut microbiota predominated by *Lactobacillus* spp. has been shown as the unique characteristic of mice on both long-term and short-term CR (27, 29). The Lactobacillus murinus CR147 strain, isolated from healthy CR mice, can enhance intestinal barrier function and reduce systemic inflammatory marker in old microbiota-colonized mice (29). Other strains, such as Lactobacillus plantarum DSM 2648, also can tightly adhere to the intestinal epithelium and abrogate bacterial translocation to mesenteric lymph nodes (45). The *L. plantarum* MB452 strain can also enhance the intestinal barrier function via adjusting the tight junction-related genes (46). Moreover, the Lactobacillus rhamnosus Gorbache Goldin strain can reduce intestinal and liver oxidative stress (47). Thus, these results support our finding that the increased abundance of *Lactobacillus* phylotypes in our CR mice may contribute to reducing the intestinal permeability and oxidative stress caused by CTX.

We also found that specific phylotypes of *Lachnospiraceae*, members of which are known as butyrate-producing bacteria (34, 48), were increased by CR and decreased by CTX. Butyrate acts as an energy substrate for the colonocytes and has a trophic effect on mucosa (49, 50). The enrichment of these *Lachnospiraceae* phylotypes might play an important role in the protection of intestinal epithelium by CR. On the other hand, the majority of *Barnesiella* phylotypes decreased upon CR. The members of *Barnesiella* were reported to be associated with systemic inflammation and oxidative DNA damage after radiotherapy (51). Reduction of this group of bacteria might be one of the reasons for the lower amount of damage caused by CTX in CR mice. The results from the current work, supported by previous findings, show that the gut microbiota improved by CR may play an important role in protecting the host from the damage caused by chemotherapy.

In conclusion, our work suggests that CR-remodeled gut microbiota mitigate the side effects of chemotherapy. Gut microbiota should have a critical role in the development of precision treatment strategies for cancer, and it will be increasingly seen as a component for next-generation cancer therapies.

## MATERIALS AND METHODS

### Animal trial and samples.

Specific-pathogen-free, 6-week-old male C57BL/6 mice (*n* = 70) were purchased from SLAC Inc. China (Shanghai, China). All mice were housed individually and randomly separated into two groups, the calorie-restricted (CR) group (*n* = 35) (fed with 70% normal chow diet of the *ad libitum*-fed mice) and the *ad libitum*-fed group of mice (*n* = 35) (see Fig. S1 in the supplemental material). The daily consumption of food in the *ad libitum*-fed group was recorded over 1 week and averaged to determine the amount of food given every day for the following week for CR. We weighed and changed food once a day for all the mice. After 28 days of calorie restriction, the CR group mice were assigned into two groups, the CR+NS group and CR+CTX group. At day A0, cyclophosphamide (CTX) (100 mg/kg of body weight) was intraperitoneally injected into CR+CTX mice, and normal saline (NS) was intraperitoneally injected into CR+NS mice (once) for a control. The same grouping was also applying to the *ad libitum*-fed mice: *ad libitum*+CTX intraperitoneally injected with CTX and *ad libitum*+NS intraperitoneally injected with normal saline (Fig. S1). To determine toxicity and efficacy, the mice were monitored routinely for weight loss and general behavior (52).

Fresh feces were collected daily. All fecal samples were stored at −80°C until analysis. Five mice from each group were sacrificed on day 28 (28d), A2d (day 2 *ad libitum*), A7d, and A10d. The blood, colon contents, colon tissues, and cecum content were collected. Mice were humanely euthanized prior to serum and tissue sample collection.

### Intestinal stem cell staining and count.

BrdU retaining assay for labeling during gut development, C57BL/6 mice (I4d) were injected intraperitoneally with BrdU (100 mg/kg of body weight; Sigma) three times daily for 2 days. Tissues were collected 7 days after BrdU administration (53). Tissue sections were then stained with an anti-BrdU polyclonal antibody (1:50 dilution; abcam), and then goat anti-chicken IgY H&L labeled with Alexa Fluor 488 (1:1,000 dilution; abcam) was used to immunofluorescent stain the BrdU-adherent stem cells(53).

### Histological analysis of colon tissues.

For histological analysis, tissues were fixed in 4% paraformaldehyde, then ethanol dehydrated, embedded in paraffin, and sectioned as described previously (54). Hematoxylin-eosin (HE) staining was performed by standard methods. Histological scoring was performed by measuring inflammation and damage as previously described (54).

All the images were taken by using a Leica CTR6000 microscope. Brightness and contrast were adjusted linearly across the entire image for any particular image.

### Intestinal permeability assay and bacterial translocation detection.

The gut barrier integrity was assessed by permeability to fluorescein isothiocyanate-dextran (FITC-dextran; Sigma). After injecting the mice for 48 h with NS or CTX at 100 mg/kg body weight, mice were forced to fast for 4 h and then orally fed with FITC-dextran at 0. 6 mg/g body weight. After 4 h, the mice were euthanized and exsanguinated by cardiac puncture. Plasma FITC levels were subsequently determined using a fluorescence spectrophotometer (485/545 nm) (22).

Mesenteric lymph nodes and spleens were aseptically removed, smashed in PBS (200 μl), and plated onto LB agar plates. After 48 h of aerobic culture, the numbers of CFU were calculated and analyzed statistically.

### Serum LBP measurement.

Blood samples were collected from the eyes and centrifuged at 12,000 × *g* for 30 min to pellet blood cells, and the serum samples were stored at −80°C until further analyses. Serum LBP was determined after a dilution of 1:1,600 using the Mouse Lipopolysaccharide Binding Protein ELISA kit (Cell Sciences, Canton, MA, USA) according to the instructions of the manufacturer.

### Microbiota DNA extraction and Illumina V3-V4 regions in 16S rRNA gene sequencing.

DNA was extracted from fecal samples at the 28d, A2d, A3d, A4d, A7d, and A10d and analyzed as previously described (55). The extracted DNA purified with the Omega Gel Extraction kit (catalog no. D2501-01; OMEGA Bio-Tek, Taiwan, China) using both physical and chemical lysis. DNA concentration and integrity were determined both visually by electrophoresis on a 1% agarose gel and spectrophotometrically by using a biodrop instrument.

The microbiota composition was assessed by Illumina targeting the V3-V4 region of the bacterial 16S rRNA gene with the primer. The primers were used to build the library by PCRs as previously described (56). PCR was performed using the following program: predenaturation at 94°C for 3 min; 22 cycles, with 1 cycle consisting of denaturation at 94°C for 30 s, annealing at 55°C for 30 s, and elongation at 72°C for 30 s; final extension at 72°C for 8 min.

For the Index PCR (attachment of dual indices and Illumina sequencing adapter using the Nextera XT Index kit), PCR program of Index PCR was the same as for Amplicon PCR except the cycle number decreased to 8. The purified products from different samples were mixed at equal ratios for sequencing using the Illumina MiSeq platform (Illumina Inc., USA).

### Bioinformatics and statistical analysis.

Both the forward and the reverse ends were cut off from the same reads at the first base for which the Q value was less than 2. All of the reads were kept when the length was more than 399 bp and the expected error was less than 0.5 (57).

High-quality sequences were clustered into OTUs (operational taxonomic units) at 97% identity by Usearch and the representative nonchimeric OTU sequences were picked by Uparse’s default (58). The number of high-quality reads was more than 10,000 for each sample. The representative sequences of each OTU were classified by the RDP classifier online, and the RDP-classified sequences were used for taxonomical assignments at 80% confidence level (59).

The tree, together with sequence abundance data, was then used for beta-diversity analysis based on weighted metric by QIIME 1.6 (60). The relative abundances of OTUs were used for principal-component analysis, multivariate analysis of variance, and redundancy analysis via Matlab R2015a (The MathWorks, Natick, MA, USA) and Canoco for Windows 4.5 (Microcomputer Power, NY, USA).

Two-way ANOVA test and Mann-Whitney test were used to test the statistical significance of the physiological and biochemical data via software SPSS 19.0 (SPSS Inc, Chicago, IL, USA). *P* values were adjusted by the method of Benjamini and Hochberg (61).

### Statistical analyses.

Redundancy analysis was introduced to identify specific bacterial phylotypes that contributed to the segregation of gut microbiota by calorie restriction and CTX. The samples from all the mice at 28 days were used to establish classification models of diet. Samples from mice on the *ad libitum*+CTX and *ad libitum*+NS on A3 day, A4 day, and A7 day were used to establish classification models of CTX.

The correlation among OTUs was calculated using the Spearman algorithm. PERMANOVA (9,999 permutations, *P* < 0.05) based on Spearman correlation coefficients was used to cluster the OTUs into coabundance groups (CAGs) using the R program.

### Accession number(s).

The 16S rRNA gene sequence information in this study has been submitted to the GenBank Sequence Read Archive database under accession number SRP166816 (https://www.ncbi.nlm.nih.gov/).

## References

[1] Schiavoni G, Sistigu A, Valentini M, Mattei F, Sestili P, Spadaro F, Sanchez M, Lorenzi S, D'Urso MT, Belardelli F, Gabriele L, Proietti E, Bracci L 2011 Cyclophosphamide synergizes with type I interferons through systemic dendritic cell reactivation and induction of immunogenic tumor apoptosis. Cancer Res 71:768–778. doi:10.1158/0008-5472.CAN-10-2788. 21156650

[2] Colleoni M, Rocca A, Sandri MT, Zorzino L, Masci G, Nole F, Peruzzotti G, Robertson C, Orlando L, Cinieri S, De Braud F, Viale G, Goldhirsch A 2002 Low-dose oral methotrexate and cyclophosphamide in metastatic breast cancer: antitumor activity and correlation with vascular endothelial growth factor levels. Ann Oncol 13:73–80. doi:10.1093/annonc/mdf013. 11863115

[3] Glode LM, Barqawi A, Crighton F, Crawford ED, Kerbel R 2003 Metronomic therapy with cyclophosphamide and dexamethasone for prostate carcinoma. Cancer 98:1643–1648. doi:10.1002/cncr.11713. 14534880

[4] Emadi A, Jones RJ, Brodsky RA 2009 Cyclophosphamide and cancer: golden anniversary. Nat Rev Clin Oncol 6:638–647. doi:10.1038/nrclinonc.2009.146. 19786984

[5] Keefe DM, Cummins AG, Dale BM, Kotasek D, Robb TA, Sage RE 1997 Effect of high-dose chemotherapy on intestinal permeability in humans. Clin Sci (Lond) 92:385–389. doi:10.1042/cs0920385. 9176038

[6] Parrilli G, Iaffaioli RV, Martorano M, Cuomo R, Tafuto S, Zampino MG, Budillon G, Bianco AR 1989 Effects of anthracycline therapy on intestinal absorption in patients with advanced breast cancer. Cancer Res 49:3689–3691. 2731182

[7] Emmenegger U, Man S, Shaked Y, Francia G, Wong JW, Hicklin DJ, Kerbel RS 2004 A comparative analysis of low-dose metronomic cyclophosphamide reveals absent or low-grade toxicity on tissues highly sensitive to the toxic effects of maximum tolerated dose regimens. Cancer Res 64:3994–4000. doi:10.1158/0008-5472.CAN-04-0580. 15173013

[8] Potten CS 1997 Epithelial cell growth and differentiation. II. Intestinal apoptosis. Am J Physiol 273:G253–G257. doi:10.1152/ajpgi.1997.273.2.G253. 9277401

[9] Keefe DM, Brealey J, Goland GJ, Cummins AG 2000 Chemotherapy for cancer causes apoptosis that precedes hypoplasia in crypts of the small intestine in humans. Gut 47:632–637. doi:10.1136/gut.47.5.632. 11034578 PMC1728102

[10] Russo F, Linsalata M, Clemente C, D'Attoma B, Orlando A, Campanella G, Giotta F, Riezzo G 2013 The effects of fluorouracil, epirubicin, and cyclophosphamide (FEC60) on the intestinal barrier function and gut peptides in breast cancer patients: an observational study. BMC Cancer 13:56. doi:10.1186/1471-2407-13-56. 23379680 PMC3575294

[11] Gibson RJ, Keefe DM 2006 Cancer chemotherapy-induced diarrhoea and constipation: mechanisms of damage and prevention strategies. Support Care Cancer 14:890–900. doi:10.1007/s00520-006-0040-y. 16604351

[12] Eutamene H, Lamine F, Chabo C, Theodorou V, Rochat F, Bergonzelli GE, Corthesy-Theulaz I, Fioramonti J, Bueno L 2007 Synergy between Lactobacillus paracasei and its bacterial products to counteract stress-induced gut permeability and sensitivity increase in rats. J Nutr 137:1901–1907. doi:10.1093/jn/137.8.1901. 17634262

[13] Heyman M, Terpend K, Menard S 2005 Effects of specific lactic acid bacteria on the intestinal permeability to macromolecules and the inflammatory condition. Acta Paediatr Suppl 94:34–36. doi:10.1080/08035320510043853. 16214764

[14] Qin HL, Zheng JJ, Tong DN, Chen WX, Fan XB, Hang XM, Jiang YQ 2008 Effect of Lactobacillus plantarum enteral feeding on the gut permeability and septic complications in the patients with acute pancreatitis. Eur J Clin Nutr 62:923–930. doi:10.1038/sj.ejcn.1602792. 17579653

[15] Loubinoux J, Mory F, Pereira IAC, Le Faou AE 2000 Bacteremia caused by a strain of Desulfovibrio related to the provisionally named Desulfovibrio fairfieldensis. J Clin Microbiol 38:931–934. 10655421 10.1128/jcm.38.2.931-934.2000 PMC86253

[16] Weglarz L, Dzierzewicz Z, Skop B, Orchel A, Parfiniewicz B, Wiśniowska B, Swiatkowska L, Wilczok T 2003 Desulfovibrio desulfuricans lipopolysaccharides induce endothelial cell IL-6 and IL-8 secretion and E-selectin and VCAM-1 expression. Cell Mol Biol Lett 8:991–1003. 14668922

[17] Stratiki Z, Costalos C, Sevastiadou S, Kastanidou O, Skouroliakou M, Giakoumatou A, Petrohilou V 2007 The effect of a bifidobacter supplemented bovine milk on intestinal permeability of preterm infants. Early Hum Dev 83:575–579. doi:10.1016/j.earlhumdev.2006.12.002. 17229535

[18] Mack DR, Michail S, Wei S, McDougall L, Hollingsworth MA 1999 Probiotics inhibit enteropathogenic E. coli adherence in vitro by inducing intestinal mucin gene expression. Am J Physiol 276:G941–G950. doi:10.1152/ajpgi.1999.276.4.G941. 10198338

[19] Chiva M, Soriano G, Rochat I, Peralta C, Rochat F, Llovet T, Mirelis B, Schiffrin EJ, Guarner C, Balanzo J 2002 Effect of Lactobacillus johnsonii La1 and antioxidants on intestinal flora and bacterial translocation in rats with experimental cirrhosis. J Hepatol 37:456–462. doi:10.1016/S0168-8278(02)00142-3. 12217598

[20] Madsen K, Cornish A, Soper P, McKaigney C, Jijon H, Yachimec C, Doyle J, Jewell L, De Simone C 2001 Probiotic bacteria enhance murine and human intestinal epithelial barrier function. Gastroenterology 121:580–591. doi:10.1053/gast.2001.27224. 11522742

[21] Resta-Lenert S, Barrett KE 2003 Live probiotics protect intestinal epithelial cells from the effects of infection with enteroinvasive Escherichia coli (EIEC). Gut 52:988–997. doi:10.1136/gut.52.7.988. 12801956 PMC1773702

[22] Viaud S, Saccheri F, Mignot G, Yamazaki T, Daillere R, Hannani D, Enot DP, Pfirschke C, Engblom C, Pittet MJ, Schlitzer A, Ginhoux F, Apetoh L, Chachaty E, Woerther PL, Eberl G, Berard M, Ecobichon C, Clermont D, Bizet C, Gaboriau-Routhiau V, Cerf-Bensussan N, Opolon P, Yessaad N, Vivier E, Ryffel B, Elson CO, Dore J, Kroemer G, Lepage P, Boneca IG, Ghiringhelli F, Zitvogel L 2013 The intestinal microbiota modulates the anticancer immune effects of cyclophosphamide. Science 342:971–976. doi:10.1126/science.1240537. 24264990 PMC4048947

[23] van Vliet MJ, Tissing WJ, Dun CA, Meessen NE, Kamps WA, de Bont ES, Harmsen HJ 2009 Chemotherapy treatment in pediatric patients with acute myeloid leukemia receiving antimicrobial prophylaxis leads to a relative increase of colonization with potentially pathogenic bacteria in the gut. Clin Infect Dis 49:262–270. doi:10.1086/599346. 19514856

[24] De Filippo C, Cavalieri D, Di Paola M, Ramazzotti M, Poullet JB, Massart S, Collini S, Pieraccini G, Lionetti P 2010 Impact of diet in shaping gut microbiota revealed by a comparative study in children from Europe and rural Africa. Proc Natl Acad Sci U S A 107:14691–14696. doi:10.1073/pnas.1005963107. 20679230 PMC2930426

[25] Mai V 2004 Dietary modification of the intestinal microbiota. Nutr Rev 62:235–242. doi:10.1111/j.1753-4887.2004.tb00045.x. 15291396

[26] Zhang CH, Zhang MH, Pang XY, Zhao YF, Wang LH, Zhao LP 2012 Structural resilience of the gut microbiota in adult mice under high-fat dietary perturbations. ISME J 6:1848–1857. doi:10.1038/ismej.2012.27. 22495068 PMC3446802

[27] Zhang C, Li S, Yang L, Huang P, Li W, Wang S, Zhao G, Zhang M, Pang X, Yan Z, Liu Y, Zhao L 2013 Structural modulation of gut microbiota in life-long calorie-restricted mice. Nat Commun 4:2163. doi:10.1038/ncomms3163. 23860099 PMC3717500

[28] Omodei D, Fontana L 2011 Calorie restriction and prevention of age-associated chronic disease. FEBS Lett 585:1537–1542. doi:10.1016/j.febslet.2011.03.015. 21402069 PMC3439843

[29] Pan F, Zhang L, Li M, Hu Y, Zeng B, Yuan H, Zhao L, Zhang C 2018 Predominant gut Lactobacillus murinus strain mediates anti-inflammaging effects in calorie-restricted mice. Microbiome 6:54. doi:10.1186/s40168-018-0440-5. 29562943 PMC5863386

[30] Blagosklonny MV 2010 Calorie restriction: decelerating mTOR-driven aging from cells to organisms (including humans). Cell Cycle 9:683–688. doi:10.4161/cc.9.4.10766. 20139716

[31] Cuervo AM, Bergamini E, Brunk UT, Droge W, Ffrench M, Terman A 2005 Autophagy and aging: the importance of maintaining “clean” cells. Autophagy 1:131–140. doi:10.4161/auto.1.3.2017. 16874025

[32] Lee C, Safdie FM, Raffaghello L, Wei M, Madia F, Parrella E, Hwang D, Cohen P, Bianchi G, Longo VD 2010 Reduced levels of IGF-I mediate differential protection of normal and cancer cells in response to fasting and improve chemotherapeutic index. Cancer Res 70:1564–1572. doi:10.1158/0008-5472.CAN-09-3228. 20145127 PMC2836202

[33] Cani PD, Bibiloni R, Knauf C, Waget A, Neyrinck AM, Delzenne NM, Burcelin R 2008 Changes in gut microbiota control metabolic endotoxemia-induced inflammation in high-fat diet-induced obesity and diabetes in mice. Diabetes 57:1470–1481. doi:10.2337/db07-1403. 18305141

[34] Louis P, Flint HJ 2009 Diversity, metabolism and microbial ecology of butyrate-producing bacteria from the human large intestine. FEMS Microbiol Lett 294:1–8. doi:10.1111/j.1574-6968.2009.01514.x. 19222573

[35] Zhao L, Zhang F, Ding X, Wu G, Lam YY, Wang X, Fu H, Xue X, Lu C, Ma J, Yu L, Xu C, Ren Z, Xu Y, Xu S, Shen H, Zhu X, Shi Y, Shen Q, Dong W, Liu R, Ling Y, Zeng Y, Wang X, Zhang Q, Wang J, Wang L, Wu Y, Zeng B, Wei H, Zhang M, Peng Y, Zhang C 2018 Gut bacteria selectively promoted by dietary fibers alleviate type 2 diabetes. Science 359:1151–1156. doi:10.1126/science.aao5774. 29590046

[36] Claesson MJ, Jeffery IB, Conde S, Power SE, O’Connor EM, Cusack S, Harris HMB, Coakley M, Lakshminarayanan B, O’Sullivan O, Fitzgerald GF, Deane J, O’Connor M, Harnedy N, O’Connor K, O’Mahony D, van Sinderen D, Wallace M, Brennan L, Stanton C, Marchesi JR, Fitzgerald AP, Shanahan F, Hill C, Ross RP, O’Toole PW 2012 Gut microbiota composition correlates with diet and health in the elderly. Nature 488:178–184. doi:10.1038/nature11319. 22797518

[37] Love RR, Leventhal H, Easterling DV, Nerenz DR 1989 Side effects and emotional distress during cancer chemotherapy. Cancer 63:604–612. doi:10.1002/1097-0142(19890201)63:3<604::AID-CNCR2820630334>3.0.CO;2-2. 2912536

[38] Iida N, Dzutsev A, Stewart CA, Smith L, Bouladoux N, Weingarten RA, Molina DA, Salcedo R, Back T, Cramer S, Dai RM, Kiu H, Cardone M, Naik S, Patri AK, Wang E, Marincola FM, Frank KM, Belkaid Y, Trinchieri G, Goldszmid RS 2013 Commensal bacteria control cancer response to therapy by modulating the tumor microenvironment. Science 342:967–970. doi:10.1126/science.1240527. 24264989 PMC6709532

[39] Bowen JM, Stringer AM, Gibson RJ, Yeoh AS, Hannam S, Keefe DM 2007 VSL#3 probiotic treatment reduces chemotherapy-induced diarrhea and weight loss. Cancer Biol Ther 6:1449–1454. 17881902 10.4161/cbt.6.9.4622

[40] Lee C, Raffaghello L, Brandhorst S, Safdie FM, Bianchi G, Martin-Montalvo A, Pistoia V, Wei M, Hwang S, Merlino A, Emionite L, de Cabo R, Longo VD 2012 Fasting cycles retard growth of tumors and sensitize a range of cancer cell types to chemotherapy. Sci Transl Med 4:124ra27. doi:10.1126/scitranslmed.3003293. PMC3608686 22323820

[41] Raffaghello L, Lee C, Safdie FM, Wei M, Madia F, Bianchi G, Longo VD 2008 Starvation-dependent differential stress resistance protects normal but not cancer cells against high-dose chemotherapy. Proc Natl Acad Sci U S A 105:8215–8220. doi:10.1073/pnas.0708100105. 18378900 PMC2448817

[42] Cho I, Blaser MJ 2012 The human microbiome: at the interface of health and disease. Nat Rev Genet 13:260–270. doi:10.1038/nrg3182. 22411464 PMC3418802

[43] Lozupone CA, Stombaugh JI, Gordon JI, Jansson JK, Knight R 2012 Diversity, stability and resilience of the human gut microbiota. Nature 489:220–230. doi:10.1038/nature11550. 22972295 PMC3577372

[44] Sekirov I, Tam NM, Jogova M, Robertson ML, Li Y, Lupp C, Finlay BB 2008 Antibiotic-induced perturbations of the intestinal microbiota alter host susceptibility to enteric infection. Infect Immun 76:4726–4736. doi:10.1128/IAI.00319-08. 18678663 PMC2546810

[45] Anderson RC, Cookson AL, McNabb WC, Kelly WJ, Roy NC 2010 Lactobacillus plantarum DSM 2648 is a potential probiotic that enhances intestinal barrier function. FEMS Microbiol Lett 309:184–192. doi:10.1111/j.1574-6968.2010.02038.x. 20618863

[46] Anderson RC, Cookson AL, McNabb WC, Park Z, McCann MJ, Kelly WJ, Roy NC 2010 Lactobacillus plantarum MB452 enhances the function of the intestinal barrier by increasing the expression levels of genes involved in tight junction formation. BMC Microbiol 10:316. doi:10.1186/1471-2180-10-316. 21143932 PMC3004893

[47] Forsyth CB, Farhadi A, Jakate SM, Tang Y, Shaikh M, Keshavarzian A 2009 Lactobacillus GG treatment ameliorates alcohol-induced intestinal oxidative stress, gut leakiness, and liver injury in a rat model of alcoholic steatohepatitis. Alcohol 43:163–172. doi:10.1016/j.alcohol.2008.12.009. 19251117 PMC2675276

[48] Louis P, Young P, Holtrop G, Flint HJ 2010 Diversity of human colonic butyrate-producing bacteria revealed by analysis of the butyryl-CoA:acetate CoA-transferase gene. Environ Microbiol 12:304–314. doi:10.1111/j.1462-2920.2009.02066.x. 19807780

[49] Bartholome AL, Albin DM, Baker DH, Holst JJ, Tappenden KA 2004 Supplementation of total parenteral nutrition with butyrate acutely increases structural aspects of intestinal adaptation after an 80% jejunoileal resection in neonatal piglets. JPEN J Parenter Enteral Nutr 28:210–223. doi:10.1177/0148607104028004210. 15291402

[50] Tappenden KA, Drozdowski LA, Thomson AB, McBurney MI 1998 Short-chain fatty acid-supplemented total parenteral nutrition alters intestinal structure, glucose transporter 2 (GLUT2) mRNA and protein, and proglucagon mRNA abundance in normal rats. Am J Clin Nutr 68:118–125. doi:10.1093/ajcn/68.1.118. 9665105

[51] Maier I, Berry DM, Schiestl RH 2014 Intestinal microbiota reduces genotoxic endpoints induced by high-energy protons. Radiat Res 181:45–53. doi:10.1667/RR13352.1. 24397477

[52] Nishihara T, Matsuda M, Araki H, Oshima K, Kihara S, Funahashi T, Shimomura I 2006 Effect of adiponectin on murine colitis induced by dextran sulfate sodium. Gastroenterology 131:853–861. doi:10.1053/j.gastro.2006.06.015. 16952554

[53] Takeda N, Jain R, LeBoeuf MR, Wang Q, Lu MM, Epstein JA 2011 Interconversion between intestinal stem cell populations in distinct niches. Science 334:1420–1424. doi:10.1126/science.1213214. 22075725 PMC3705713

[54] Maslowski KM, Vieira AT, Ng A, Kranich J, Sierro F, Yu D, Schilter HC, Rolph MS, Mackay F, Artis D, Xavier RJ, Teixeira MM, Mackay CR 2009 Regulation of inflammatory responses by gut microbiota and chemoattractant receptor GPR43. Nature doi:10.1038/nature08530. PMC3256734 19865172

[55] Godon JJ, Zumstein E, Dabert P, Habouzit F, Moletta R 1997 Molecular microbial diversity of an anaerobic digestor as determined by small-subunit rDNA sequence analysis. Appl Environ Microbiol 63:2802–2813. 9212428 10.1128/aem.63.7.2802-2813.1997 PMC168577

[56] Illumina Inc. 2013 16S metagenomic sequencing library preparation. Preparing 16S ribosomal RNA gene amplicons for the Illumina MiSeq system. Part 15044223. Rev. B. Illumina Inc, San Diego, CA.

[57] Edgar RC 2010 Search and clustering orders of magnitude faster than BLAST. Bioinformatics 26:2460–2461. doi:10.1093/bioinformatics/btq461. 20709691

[58] Edgar RC 2013 UPARSE: highly accurate OTU sequences from microbial amplicon reads. Nat Methods 10:996–998. doi:10.1038/nmeth.2604. 23955772

[59] Cole JR, Wang Q, Cardenas E, Fish J, Chai B, Farris RJ, Kulam-Syed-Mohideen AS, McGarrell DM, Marsh T, Garrity GM, Tiedje JM 2009 The Ribosomal Database Project: improved alignments and new tools for rRNA analysis. Nucleic Acids Res 37:D141–D145. doi:10.1093/nar/gkn879. 19004872 PMC2686447

[60] Caporaso JG, Kuczynski J, Stombaugh J, Bittinger K, Bushman FD, Costello EK, Fierer N, Pena AG, Goodrich JK, Gordon JI, Huttley GA, Kelley ST, Knights D, Koenig JE, Ley RE, Lozupone CA, McDonald D, Muegge BD, Pirrung M, Reeder J, Sevinsky JR, Turnbaugh PJ, Walters WA, Widmann J, Yatsunenko T, Zaneveld J, Knight R 2010 QIIME allows analysis of high-throughput community sequencing data. Nat Methods 7:335–336. doi:10.1038/nmeth.f.303. 20383131 PMC3156573

[61] Benjamini Y, Drai D, Elmer G, Kafkafi N, Golani I 2001 Controlling the false discovery rate in behavior genetics research. Behav Brain Res 125:279–284. doi:10.1016/S0166-4328(01)00297-2. 11682119

