# Supplementary figures and images for: A More Robust Gut Microbiota in Calorie-Restricted Mice Is Associated with Attenuated Intestinal Injury Caused by the Chemotherapy Drug Cyclophosphamide
Source: mBio. 2019 Mar 12;10(2):e02903-18. doi: 10.1128/mBio.02903-18 (PMC6414708; doi:10.1128/mBio.02903-18)

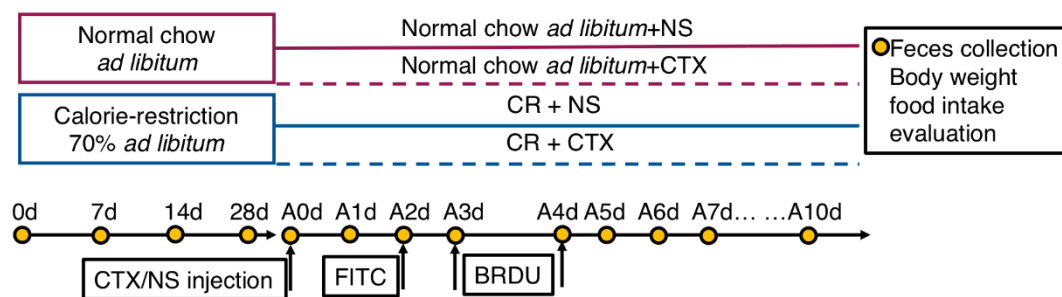

Supplement: FIG S1 [file mBio.02903-18-sf001.pdf]

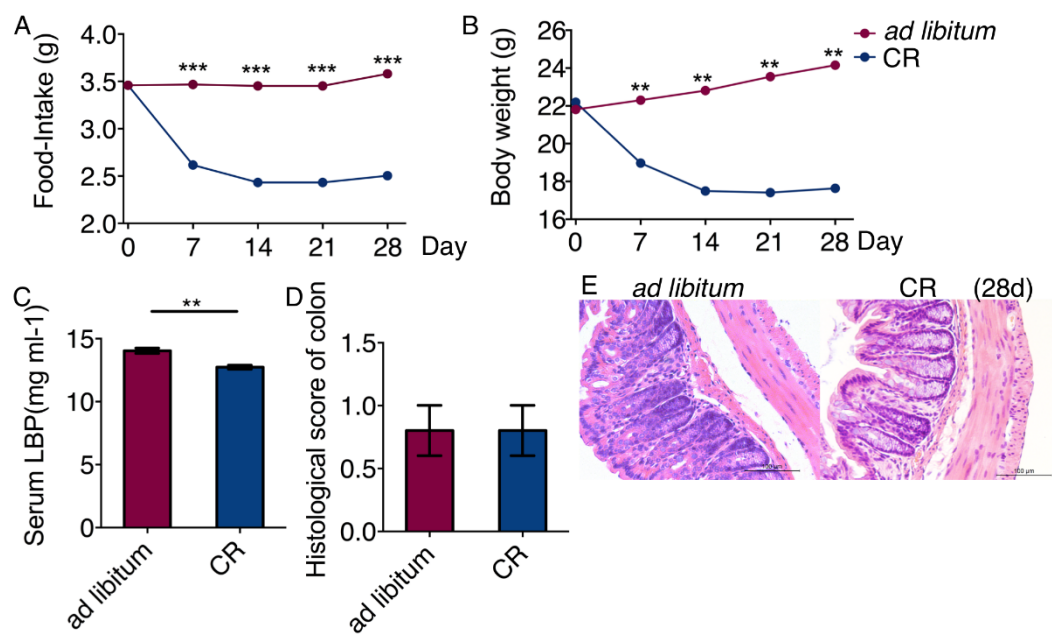

Supplement: FIG S2 [file mBio.02903-18-sf002.pdf]

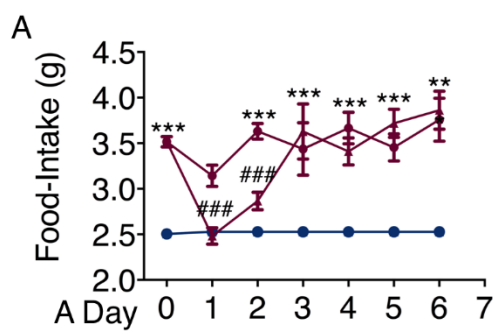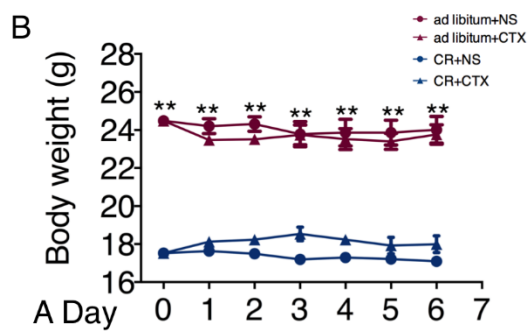

Supplement: FIG S3 [file mBio.02903-18-sf003.pdf]

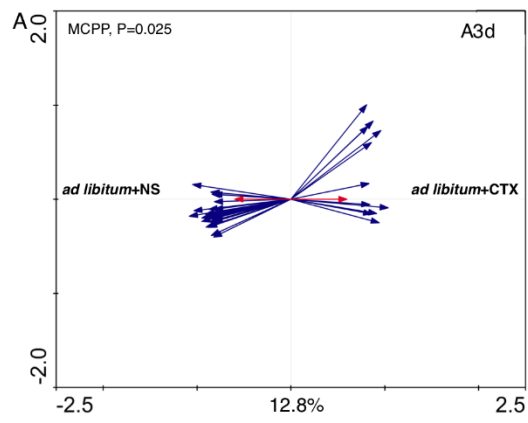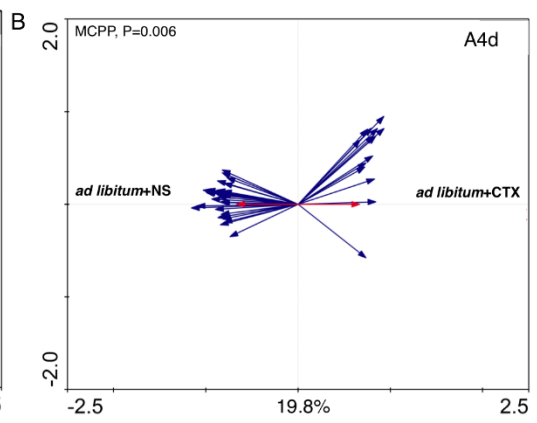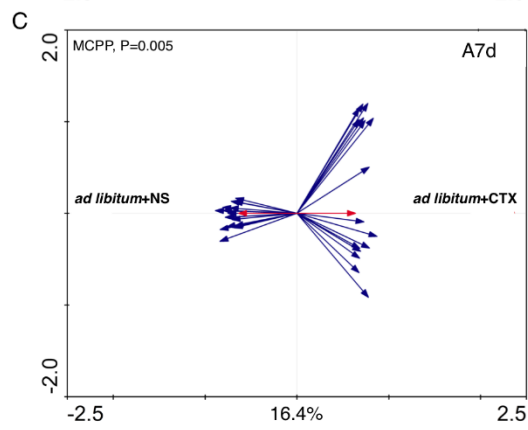

Supplement: FIG S5 [file mBio.02903-18-sf005.pdf]

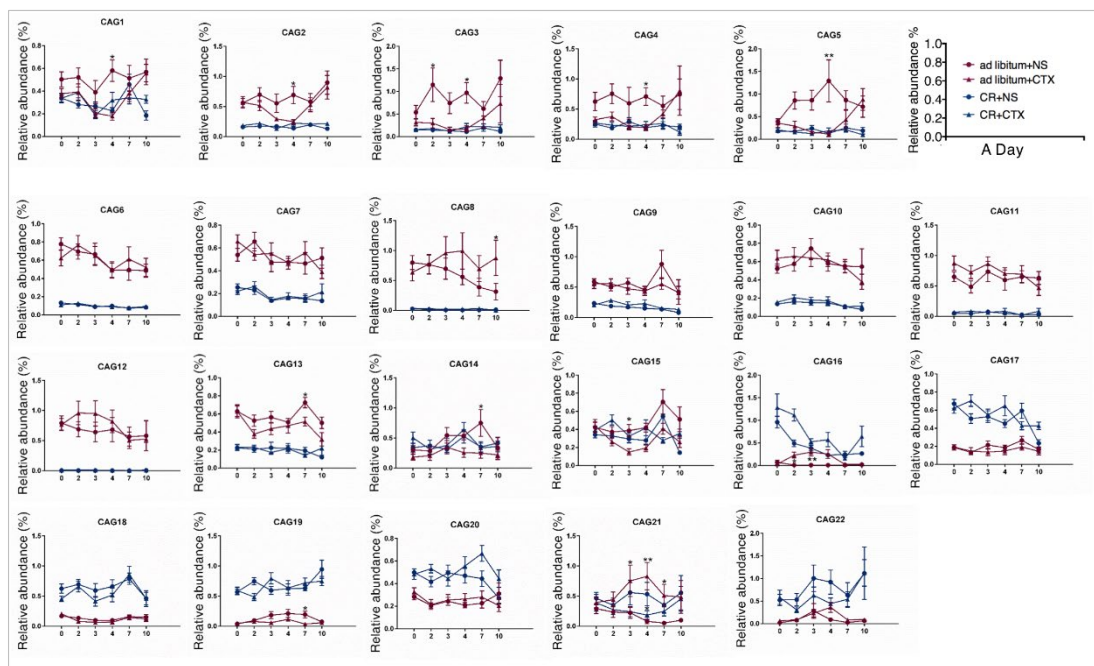

Supplement: FIG S6 [file mBio.02903-18-sf006.pdf]
