# Supplementary material for: A More Robust Gut Microbiota in Calorie-Restricted Mice Is Associated with Attenuated Intestinal Injury Caused by the Chemotherapy Drug Cyclophosphamide
Source: mBio. 2019 Mar 12;10(2):e02903-18. doi: 10.1128/mBio.02903-18 (PMC6414708; doi:10.1128/mBio.02903-18)
Supplement: TABLE S1 [file mBio.02903-18-st001.pdf]

Table S1 The effects of microbiota structure by CTX in *ad libitum* and CR group.

	A0d	A2d	A3d	A4d	A7d	A10d
ad libitum+NS VS. *ad libitum*+CTX	0.1557	0.0878	0.0283	0.0008	0.0135	0.4578
CR+NS VS. CR+CTX	0.6114	0.0173	0.227	0.5187	0.6621	0.3415
