# Supplementary material for: A More Robust Gut Microbiota in Calorie-Restricted Mice Is Associated with Attenuated Intestinal Injury Caused by the Chemotherapy Drug Cyclophosphamide
Source: mBio. 2019 Mar 12;10(2):e02903-18. doi: 10.1128/mBio.02903-18 (PMC6414708; doi:10.1128/mBio.02903-18)
Supplement: FIG S4 [file mBio.02903-18-sf004.pdf]

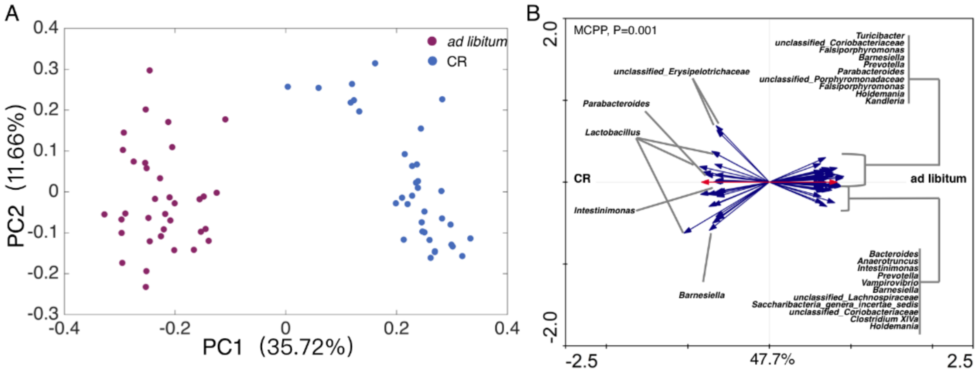
A
ad libitum
CR
PC2 (11.66%)
PC1 (35.72%)
0.4
0.3
0.2
0.1
0
-0.1
-0.2
-0.3
-0.4
-0.2
0
0.2
0.4
B
MCPP, P=0.001
2.0
-2.0
-2.5
47.7%
2.5
CR
ad libitum
unclassified_Erysipelotrichaceae
Parabacteroides
Lactobacillus
Intestinimonas
Barnesiella
Turicibacter
unclassified_Coriobacteriaceae
Falsiporphyromonas
Barnesiella
Prevotella
Parabacteroides
unclassified_Porphyromonadaceae
Falsiporphyromonas
Holdemania
Kandleria
Bacteroides
Anaerotruncus
Intestinimonas
Prevotella
Vampirovibrio
Barnesiella
unclassified_Lachnospiraceae
Saccharibacteria_genera_incertae_sedis
unclassified_Coriobacteriaceae
Clostridium XIVa
Holdemania
